# Silicone Plate for the Prevention of Postoperative Adhesions in Patients with Asherman Syndrome

**DOI:** 10.1155/2019/5420837

**Published:** 2019-11-21

**Authors:** Maho Miyagi, Keiko Mekaru, Sugiko Oishi, Chiaki Urasoe, Kozue Akamine, Yoichi Aoki

**Affiliations:** Department of Obstetrics and Gynaecology, Graduate School of Medicine, University of the Ryukyus, 207 Uehara, Nishihara, Okinawa 903-0215, Japan

## Abstract

**Purpose:**

Re-adhesion rates following hysteroscopic adhesiolysis have remained high. Accordingly, we present a case involving temporary placement of an intrauterine silicone plate to prevent re-adhesions following hysteroscopic adhesiolysis in a 36-year-old woman with Asherman syndrome.

**Methods:**

After hysteroscopic adhesiolysis, a silicone plate molded to the uterine cavity's shape was inserted into the uterine cavity and left in place for 1 month.

**Results:**

The patient had a history of endometrial curettage for endometrial polyps. After the procedure, she developed amenorrhea and experienced infertility for 5 years despite four cycles of *in vitro* fertilization and embryo transfer. Following admission to our hospital, hysteroscopic examination showed a wide area of intrauterine adhesions for which hysteroscopic adhesiolysis and silicone plate insertion were performed. The silicone plate was removed after 1 month. No intrauterine adhesions were observed during the subsequent hysteroscopic examination. After a thawed embryo transfer, the patient became pregnant and delivered a healthy baby.

**Conclusions:**

A silicon plate may be considered a useful tool for preventing re-adhesion following hysteroscopic adhesiolysis without serious complications.

## 1. Introduction

Intrauterine adhesions, such as those observed in patients with Asherman syndrome, can lead to embryo implantation disorders, causing infertility and recurrent miscarriages. This is believed to be due to endometrial damage, often caused by endometrial curettage. Specifically, each procedure carries a 16% risk of intrauterine adhesion that increases with the number of procedures performed [1]. Severe intrauterine adhesions could lead to uterine amenorrhea. Therefore, patients who plan to bear children should undergo hysteroscopic adhesiolysis. However, this procedure carries a high re-adhesion rate, reaching 62.5% in severe cases [2]. Even without severe adhesions, the re-adhesion rate following adhesiolysis is 27.2% in patients with Asherman syndrome [3]. Although an intrauterine device (IUD) is traditionally placed to prevent postoperative adhesions, its effects remain insufficient. In the field of otorhinolaryngology, medical silicone plates are being used to prevent adhesions during tympanoplasty. Here, we present a case involving temporary intrauterine placement of a silicone plate to prevent re-adhesions with successful outcomes, accompanied by a literature review.

## 2. Case Summary

Our patient was a 36-year-old G0P0 female with an unremarkable medical history except for a 5-year history of infertility. The patient sought infertility treatment from a previous physician when she was 31 years old and subsequently underwent endometrial curettage for endometrial polyps. After the procedure, she developed amenorrhea with small menstrual blood loss despite Kaufmann therapy. The patient then underwent four cycles of *in vitro* fertilization and embryo transfer. Though biochemical pregnancy was confirmed once, the patient was referred to our department for further examination and treatment.

**Figure 1:**
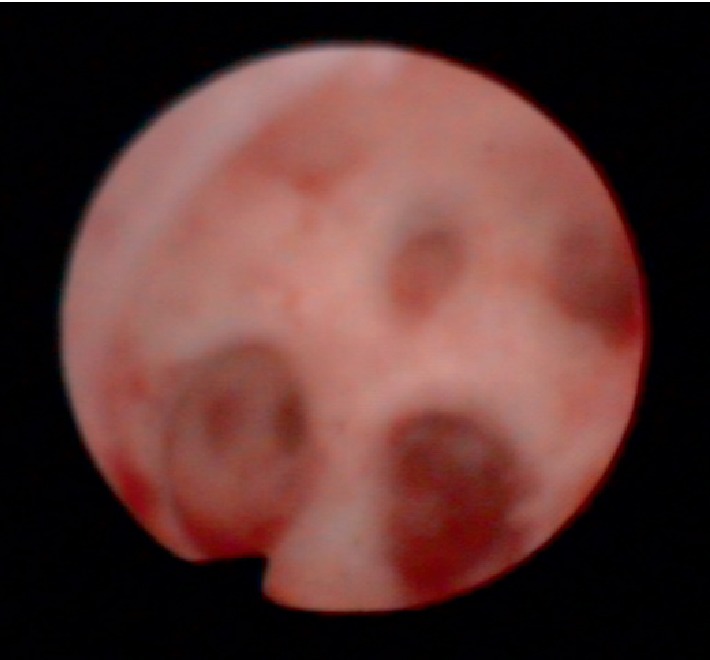
Hysteroscopic examination.

A hysteroscopic examination performed at our department confirmed a wide area of intrauterine adhesions (Figure 1), which led to a diagnosis of Asherman syndrome. In the present case, Asherman syndrome severity was evaluated using the European Society of Gynecological Endoscopy classification of intrauterine adhesions based on hysteroscopic findings. Due to their honeycomb-like appearance, the intrauterine adhesions were classified as stage III multiple dense adhesions (moderate).

### 2.1. Honeycomb-Like Adhesions and Fibrosis can be Observed in the Uterine Cavity

**Figure 2:**
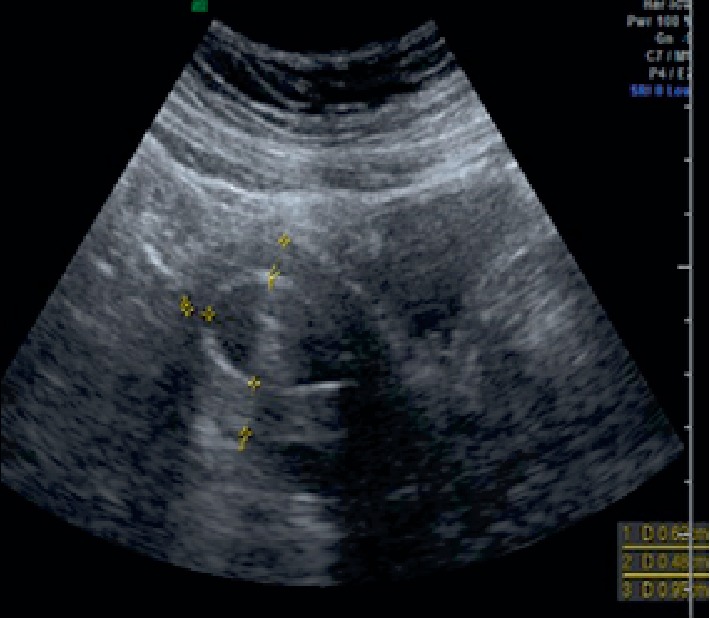
Transabdominal ultrasound was used to measure the balloon size.

**Figure 3:**
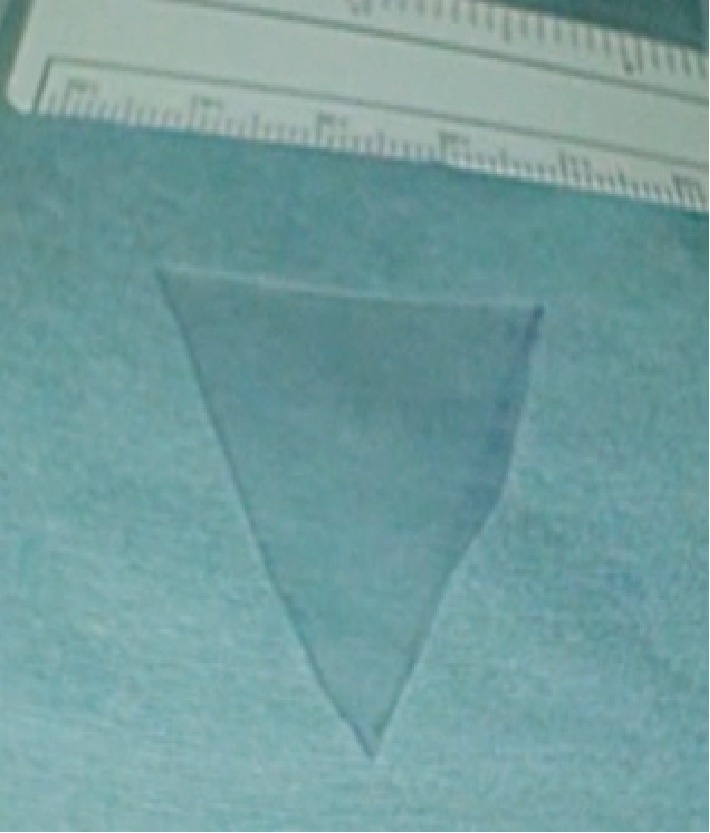
The silicone plate (200 × 150 mm silicone sheet with a thickness of 0.5 mm; Koken Co., Ltd. Bunkyo ward, Tokyo, Japan) was molded into an inverted triangle, comparable to the shape of the uterine cavity.

**Figure 4:**
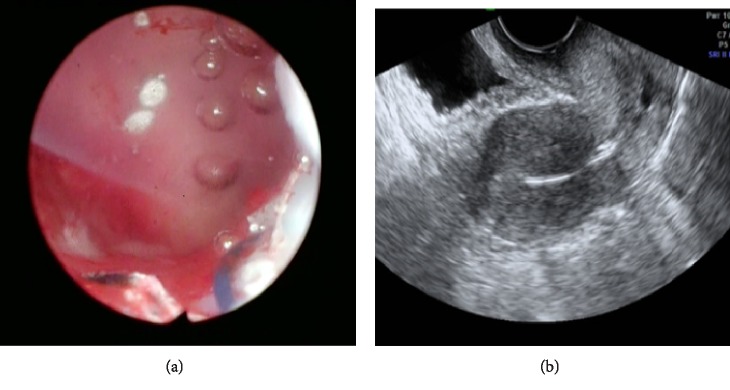
Hysteroscopic (a) and transabdominal ultrasound (b) findings for the intrauterine placement of the silicone plate.

**Figure 5:**
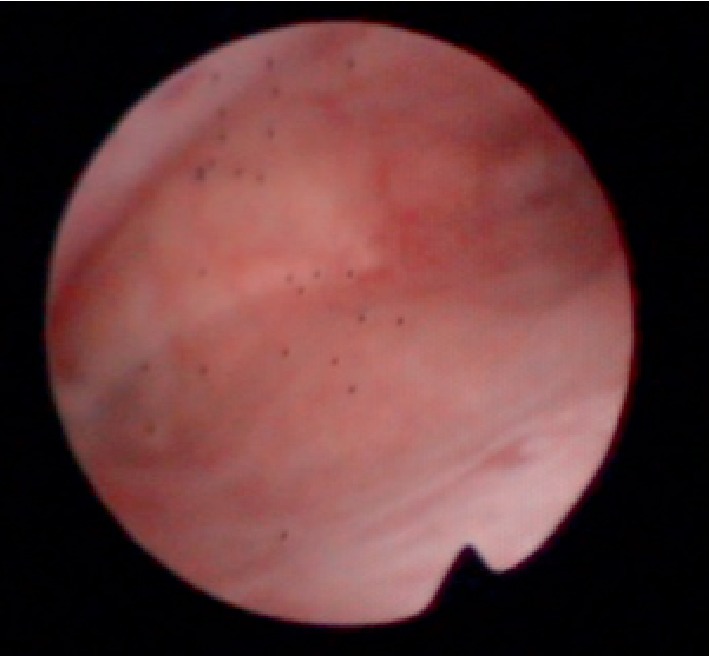
Hysteroscopic findings following silicone plate removal.

Hysteroscopic adhesiolysis was subsequently performed under general anesthesia. Initially, a 14-F urinary catheter with a balloon was inserted into the uterine cavity, after which 5 mL of saline was slowly injected into the balloon. Following blunt adhesiolysis with the balloon, partially tough, cord-like tissues were resected using an electric scalpel. Thereafter, a urinary catheter was inserted into the uterine cavity and an additional 5 mL of saline was injected. The balloon's transverse and longitudinal diameters were measured using ultrasound with the information obtained used to mold the silicone plate (Figure 2). The transverse diameter of the balloon was 3 cm, while the longitudinal diameter from the fundus to the internal ostium in the sagittal section was 3.6 cm. The silicone plate had the following characteristics: 200 × 150 mm silicone sheet with a thickness of 0.5 mm (Koken Co., Ltd. Bunkyo ward, Tokyo, Japan). The plate was molded into an inverted triangle, resembling the previously measured uterine cavity's shape (Figure 3). A nylon thread was sewn to the tail of the silicone plate to facilitate traction and removal. Finally, the silicon plate was inserted using transabdominal ultrasound and placenta forceps to match the uterine cavity's shape. The position of the inserted silicone plate was confirmed through hysteroscopy and ultrasound (Figure 4). The patient received one preoperative intravenous administration of antibiotics (1 g viccillin/administration), followed by continuous administration of the same antibiotics until postoperative day 1 (1 g viccillin/8 h) to prevent infection. The patient had an uneventful postoperative course and was discharged on postoperative day 2. She began hormone replacement therapy (HRT) 1 week post-surgery. No signs of infection were observed, and the silicone plate was removed after 1 month. The patient continued to receive two cycles of HRT. No intrauterine adhesions were found during the subsequent hysteroscopic examination (Figure 5).

*In vitro* fertilization began 9 weeks post-surgery. After a short protocol which was used for ovarian stimulation, five oocytes were retrieved under local anesthesia. Four embryos were obtained through insemination, and four day 2 early cleavage-stage embryos were frozen. Subsequently, one thawed 4-cell embryo was transferred under HRT, and the patient became pregnant.

Labor was induced with oxytocin at 41 weeks and 0 days of gestation. Due to weak uterine contractions and arrested labor, an emergency cesarean section was performed at 41 weeks and 2 days of gestation.

The infant was a male weighing 3070 g with an Apgar score of 8 at both 1 and 5 min. Umbilical cord blood gas analysis showed a pH of 7.184. No intrauterine adhesions or placenta accreta were observed intraoperatively. The patient had a good postoperative course and was discharged on postoperative day 7.

The patient provided written informed consent before surgery. This study was conducted according to the principles stated in the 1964 Declaration of Helsinki, and all subsequent revisions, and was approved by the Institutional Review Board of our university (approval number: 941).

## 3. Discussion

Asherman syndrome, which refers to the presence of partial or complete adhesions in the uterine cavity, is caused by damage to the endometrium following endometrial curettage, endometritis, transcervical resection of uterine myoma, enucleatic myomectomy reaching the endometrium intraoperatively, or a cesarean section. Considering that such adhesions can be responsible for uterine amenorrhea, infertility, and recurrent miscarriages, women with this condition who wish to bear children require appropriate treatment. Although hysteroscopic adhesiolysis of the uterine cavity has been generally performed, postoperative re-adhesion rates have remained at 27.2–62.5% [2, 3].

An earlier report had addressed this issue by temporarily inserting a medical silicone plate into the uterus to prevent adhesions [4], indicating its utility. In the field of otorhinolaryngology, medical silicone plates are also being used *in vivo* to prevent adhesions during tympanoplasty. Although our institution had previously practiced the insertion of IUDs to prevent postoperative adhesions, re-adhesions were still observed in a subset of cases. Hysteroscopic findings indicated that re-adhesion occurred on the lateral margins or fundus of the uterus, which are not in contact with the IUD. Accordingly, an IUD may have too small an area to cover the entire uterine cavity, unlike a silicone plate.

Table 1 summarizes recent prospective randomized studies investigating the role of an IUD, Foley catheter, and uterine balloon in preventing postoperative re-adhesion. Some studies have suggested that several anti-adhesion therapies may offer a potential benefit for decreasing postoperative re-adhesion in women undergoing operative hysteroscopy.

However, no strategies have been strongly recommended to decrease the recurrence rate of intrauterine adhesions [9].

One study involving Foley catheter insertion in 25 patients achieved a pregnancy rate of 33.9%, as well as a menstrual resumption rate of 81%. However, due to uterine perforation in two cases [10], this technique's safety and efficacy remains uncertain.While no uterine perforation had been in the present case, the main concern with silicone plate placement is infection. Although silicone can be placed *in vivo*, leaving it in place for 1 month increases the patient's susceptibility to endometritis or pelvic peritonitis due to increased risk of infection. To address this issue, prophylactic antibiotic administration should be considered. In the present case, prophylactic antibiotics had been administered preoperatively and 1 day postoperatively. Unlike in previous reports, our patient did not receive long-term antibiotic administration. Nonetheless, no serious complications, including infection and uterine perforation, had been observed.

During pregnancy following treatment for Asherman syndrome, incidences of premature delivery, intrauterine growth retardation, and placenta accreta had been reported [11, 12]. A 2010 review by Deans et al. found that premature delivery occurred in nearly half of the 696 cases previously treated for adhesiolysis. Additionally, the authors reported 17 cases of placental malposition, such as placenta accreta and placenta previa, and two cases of uterine rupture [11]. Due to the risk for such serious complications, pregnancy and delivery must be managed carefully, and patients must have sufficient understanding of the aforementioned risks. For severe Asherman syndrome cases, pregnancy and delivery must be managed carefully, with detailed informed consent from the patients. The present case experienced a normal pregnancy except for a short period of vaginal bleeding wherein the patient was hospitalized due to a threatened miscarriage. Finally, a healthy infant was delivered with no intrauterine adhesions or placental malposition.

## 4. Conclusion

A silicon plate may be considered a useful tool for preventing re-adhesion following hysteroscopic adhesiolysis without serious complications.

## Figures and Tables

**Table 1:** Studies investigating the role of an intrauterine devicefoley catheter, and uterine balloon in preventing intrauterine adhesion formation after hysteroscopic surgery.

	Study design	Devices	Recurrence rate	**** *p*
Gan et al. (2017) [5]	Prospective randomized	Foley catheter + freeze-dried amnion graft	28% (11/40)	0.24
		Foley catheter only	40%(16/40)	
Wang et al. (2016) [6]	Prospective randomized	Amniotic scaffold balloon	21% (6/29)	0.25
		Foley catheter only	36% (10/28)	
Xiao et al. (2015) [7]	Prospective randomized	Auto-crosslinked hyaluronic acid gel + foley catheter	13% (7/55)	0.0006
		Foley catheter only	38% (21/56)	
Lin et al. (2015) [8]	Prospective randomized	Heart-shaped balloon	30% (25/82)	NS
		Intrauterine device	35% (28/80)	
